# Supplementary material for: Molecular Dynamics Study of the Green Solvent Polyethylene Glycol with Water Impurities
Source: Molecules. 2024 Apr 30;29(9):2070. doi: 10.3390/molecules29092070 (PMC11085543; doi:10.3390/molecules29092070)
Supplement: Supplementary file 1 [file molecules-29-02070-s001.zip › molecules-2969611-supplementary.pdf]

Table S1: Number composition of simulated H-[O-CH_2-CH_2]$_n$-OH-water systems.

	w_{water}			
n	0.001	0.005	0.010	0.020
		Diethylene Glycol		
2	994	971	944	893
H_2O	6	29	56	107
		Tetraethylene glycol		
4	989	949	902	820
H_2O	11	51	98	180
		Hexaethylene glycol		
6	492	464	432	379
H_2O	8	36	68	121
		PEG200		
2	34	33	31	28
3	220	211	200	182
4	316	303	288	261
5	244	233	222	201
6	128	122	116	105
7	47	45	42	38
H_2O	11	53	101	185

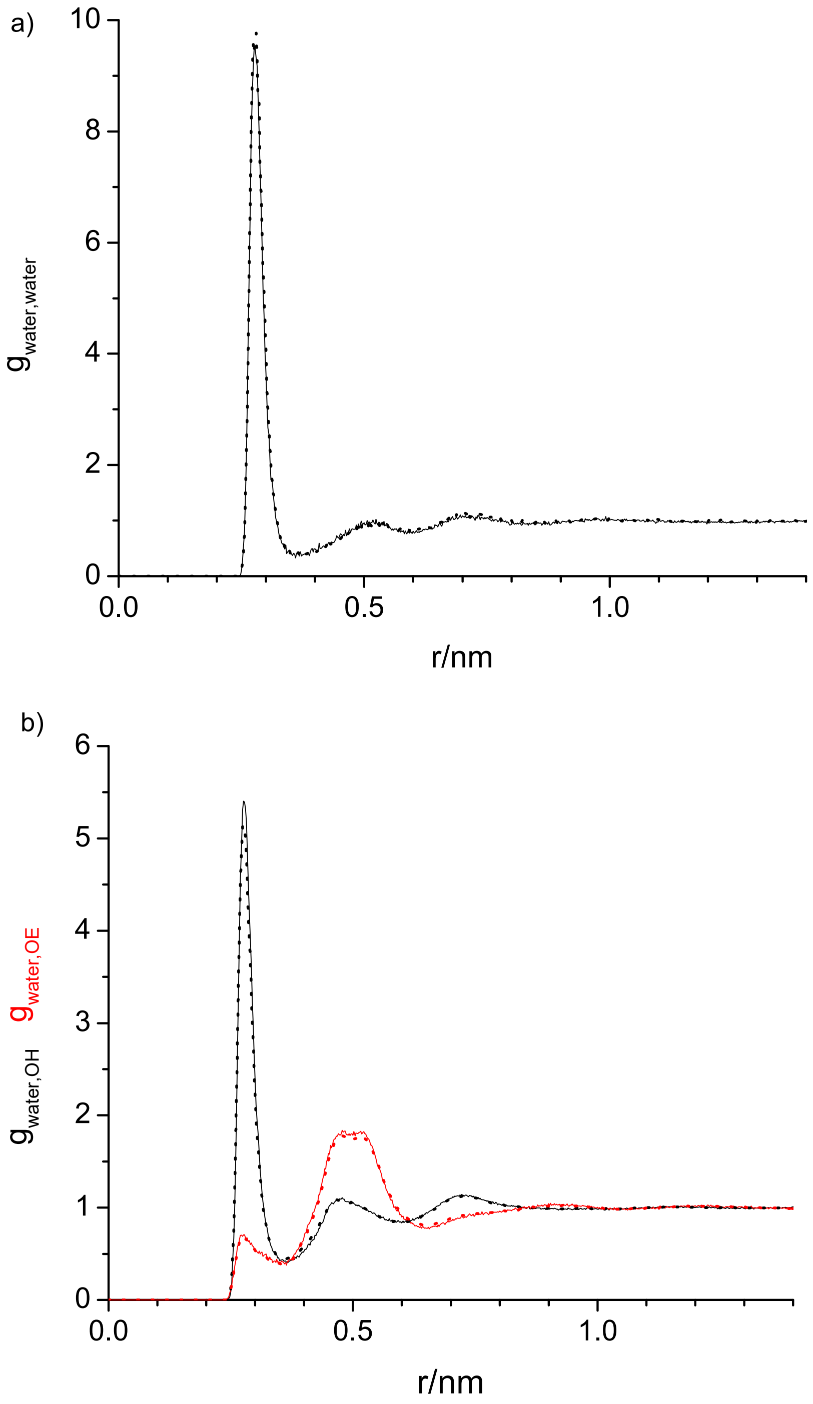

Figure S1. Radial distribution functions of water oxygen with (a) water oxygen as well as (b) oligomer hydroxy oxygen (black) and ether oxygen (red) for 0.02 mass fraction of water in diethylene glycol obtained with the water force fields TIP4P/2005 in combination with OPLS forcefield (solid) and the modified OPLS force field (dotted).

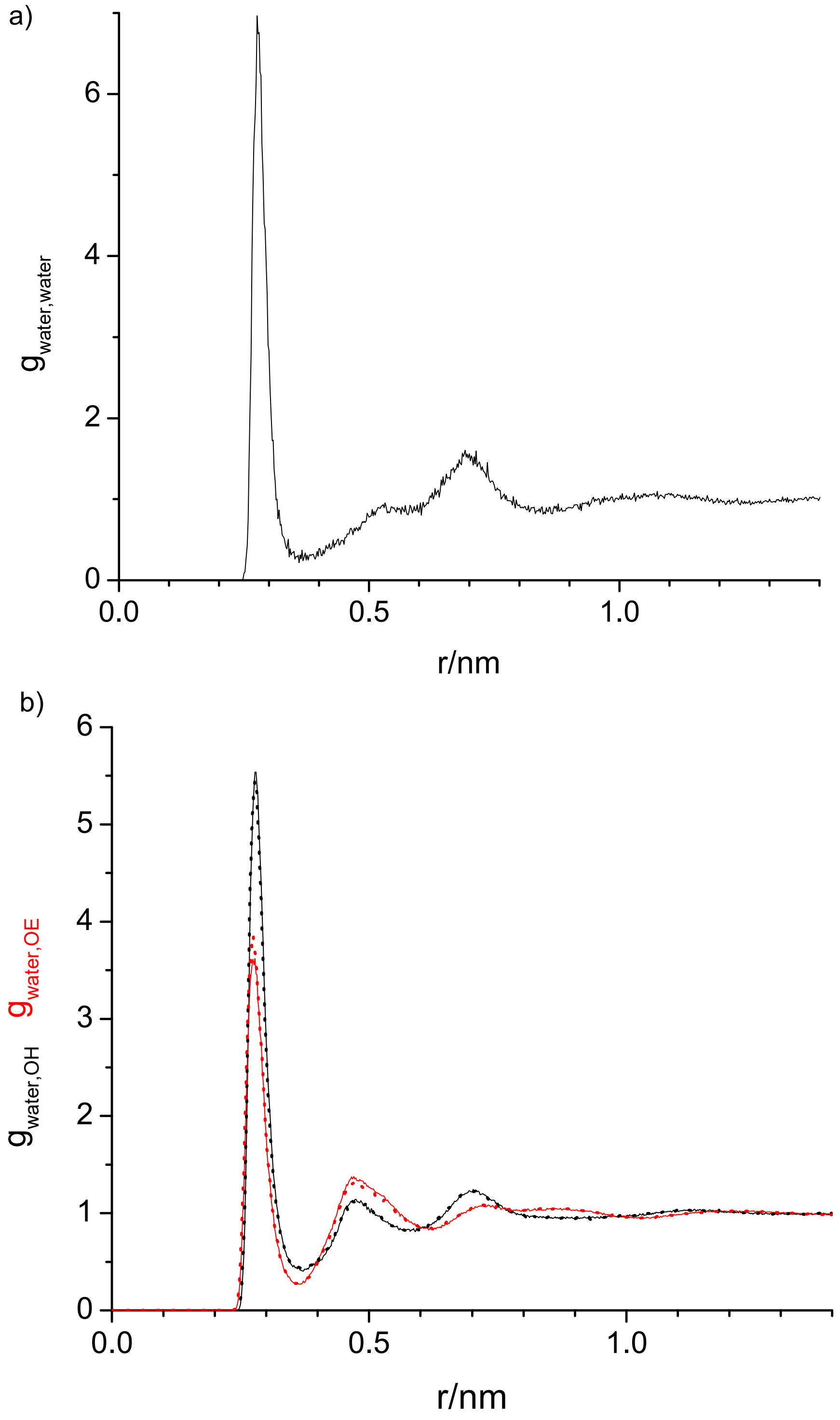

Figure S2. Radial distribution functions of water oxygen with (a) water oxygen as well as (b) oligomer hydroxy oxygen (black) and ether oxygen (red) for 0.02 mass fraction of water in tetraethylene glycol obtained with the water force fields TIP4P/2005 in combination with OPLS forcefield (solid) and the modified OPLS force field (dotted).

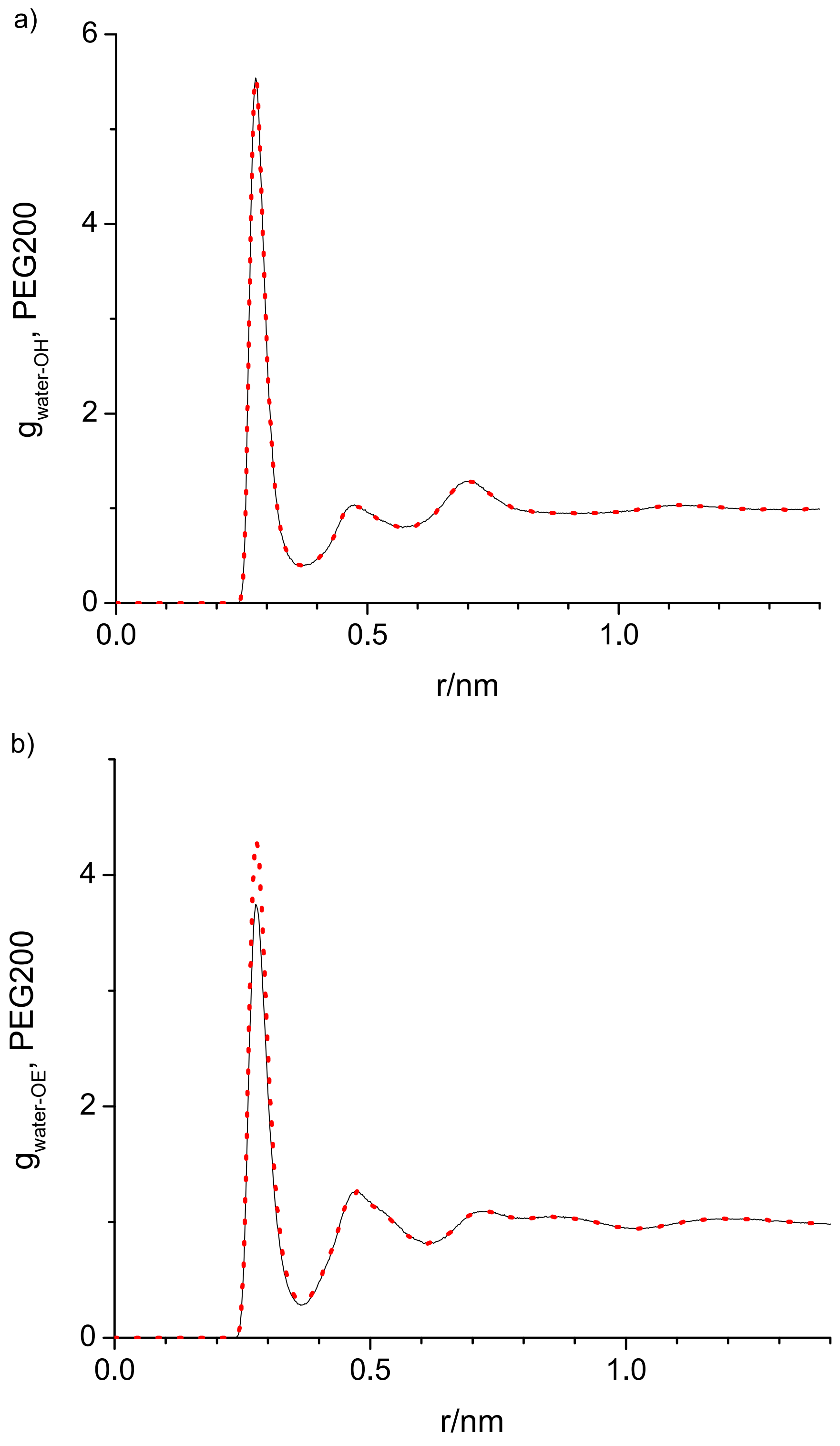

Figure S3. Calculated (solid black line) and simulated (dotted red line) radial distribution functions of water with (a) hydroxy oxygen as well as (b) ether oxygen for 0.02 mass fraction of water in PEG200 obtained with the water force fields SPC/E in combination with OPLS forcefield.

Kinetic Theory of Gases

For two water molecules to engage in hydrogen bonding, they must come in contact, i.e., collide. The number of collisions, dN_{coll}, for water behaving as an ideal gas moving through the simulation box in straight trajectories of random direction at average speed of

$$\langle u \rangle = \left(\frac{8RT}{\pi M}\right)^{1/2} \tag{S1}$$

where T is the temperature in Kelvin (328 K in this case) and M the molar mass of water, is calculated to be $dN_{\text{coll}} = 3.12 \times 10^{-4}$ using equation S2

$$dN_{\text{coll}} = \rho\sigma\langle u \rangle dt \tag{S2}$$

In equation S2, ρ is the number density (for PEG200 with $w_{\text{water}} = 0.020$ there were 185 water molecules in a cubic box of length 6.42×10^{-9} m) and σ is the collision cross section of water = 3.59×10^{-19} m^2 calculated from the van der Waals radius of 1.69×10^{-10} m.[1] The hydrogen bonding numbers from the MD simulations were obtained from averaging the counts across the recorded frames from the MD simulation where each frame is an image from the last simulation time increment, dt, of 2×10^{-15} s. The corresponding value for n_{HB}/n_{water} is then 1.68×10^{-6}.

Table S2: Simulation results at 328K for number of hydrogen bonds per number of water molecules

		w_{water}				w_{water}			
OPLS	model[a]	0.001	0.005	0.010	0.020	0.001	0.005	0.010	0.020
		Water with oligomer OH				Water with water			
		Diethylene glycol				Diethylene glycol			
Unmod	SPC/E	2.511	2.458	2.397	2.280	0.009	0.045	0.083	0.136
Unmod	TIP4P	2.493	2.444	2.397	2.320	0.004	0.034	0.063	0.138
Mod	SPC/E	2.310	2.251	2.208	2.091	0.006	0.048	0.086	0.100
Mod	TIP4P	2.299	2.271	2.232	2.159	0.008	0.032	0.061	0.097
		Tetraethylene glycol				Tetraethylene glycol			
Unmod	SPC/E	1.515	1.510	1.488	1.448	0.004	0.025	0.054	0.110
Unmod	TIP4P	1.535	1.508	1.498	1.472	0.003	0.020	0.043	0.086
Mod	SPC/E	1.544	1.515	1.494	1.443	0.005	0.031	0.062	0.127
Mod	TIP4P	1.581	1.566	1.548	1.508	0.006	0.024	0.047	0.097
		Hexaethylene glycol				Hexaethylene glycol			
Unmod	SPC/E	1.074	1.074	1.065	1.044	0.003	0.018	0.039	0.079
Unmod	TIP4P	1.072	1.049	1.053	1.037	0.003	0.012	0.032	0.063
Mod	SPC/E	0.627	0.635	0.624	0.611	0.005	0.029	0.059	0.124
Mod	TIP4P	0.625	0.631	0.625	0.620	0.004	0.020	0.040	0.085
		PEG200				PEG 200			
Unmod	SPC/E	1.397	1.389	1.379	1.356	0.005	0.020	0.043	0.087
Unmod	TIP4P	1.382	1.396	1.382	1.371	0.004	0.020	0.032	0.068
Mod	SPC/E	1.323	1.302	1.297	1.268	0.005	0.023	0.048	0.105
Mod	TIP4P	1.337	1.347	1.332	1.321	0.004	0.019	0.038	0.078
		Water with oligomer ether group				Sum of all contributions			
		Diethylene glycol				Diethylene glycol			
Unmod	SPC/E	0.141	0.145	0.139	0.156	2.661	2.648	2.619	2.572
Unmod	TIP4P	0.140	0.140	0.138	0.118	2.637	2.618	2.598	2.576
Mod	SPC/E	0.107	0.105	0.104	0.171	2.423	2.404	2.398	2.362
Mod	TIP4P	0.102	0.099	0.098	0.123	2.409	2.402	2.391	2.379
		Tetraethylene glycol				Tetraethylene glycol			
Unmod	SPC/E	1.171	1.129	1.101	1.047	2.690	2.664	2.643	2.605
Unmod	TIP4P	1.092	1.087	1.065	1.026	2.630	2.615	2.606	2.584
Mod	SPC/E	0.990	0.976	0.941	0.881	2.539	2.522	2.497	2.451
Mod	TIP4P	0.913	0.889	0.882	0.845	2.500	2.479	2.477	2.450
		Hexaethylene glycol				Hexaethylene glycol			
Unmod	SPC/E	1.587	1.560	1.530	1.472	2.664	2.652	2.634	2.595
Unmod	TIP4P	1.542	1.564	1.511	1.476	2.617	2.625	2.596	2.576
Mod	SPC/E	1.734	1.694	1.656	1.575	2.366	2.358	2.339	2.310
Mod	TIP4P	1.768	1.744	1.719	1.657	2.397	2.395	2.384	2.362
		PEG200				PEG200			
Unmod	SPC/E	1.397	1.389	1.379	1.356	2.799	2.798	2.801	2.799
Unmod	TIP4P	1.382	1.396	1.382	1.371	2.768	2.812	2.796	2.810
Mod	SPC/E	1.323	1.302	1.297	1.268	2.651	2.627	2.642	2.641
Mod	TIP4P	1.337	1.347	1.332	1.321	2.678	2.713	2.702	2.720

[a] The specific TIP4P model used was TIP4P/2005

Table S3: Simulation results at 328K for number of oligomer-oligomer hydrogen bonds per number of ethylene glycol oligomer molecules

		w_{water}					w_{water}				
OPLS	model[a]	0^2	0.001	0.005	0.010	0.020	0^2	0.001	0.005	0.010	0.020
		Intramolecular OH-OH					Intramolecular OH-OE				
		Diethylene glycol					Diethylene glycol				
Unmod	SPC/E	0.007	0.007	0.008	0.007	0.008	0.028	0.027	0.028	0.027	0.028
Unmod	TIP4P	0.007	0.008	0.008	0.008	0.007	0.028	0.027	0.028	0.028	0.028
Mod	SPC/E	0.019	0.020	0.019	0.020	0.020	0.055	0.055	0.054	0.053	0.052
Mod	TIP4P	0.019	0.021	0.019	0.020	0.019	0.055	0.055	0.055	0.056	0.053
		Tetraethylene glycol					Tetraethylene glycol				
Unmod	SPC/E	0.070	0.055	0.057	0.057	0.050	0.156	0.159	0.161	0.165	0.147
Unmod	TIP4P	0.070	0.061	0.060	0.052	0.052	0.156	0.160	0.166	0.154	0.144
Mod	SPC/E	0.122	0.121	0.119	0.110	0.110	0.332	0.316	0.328	0.314	0.309
Mod	TIP4P	0.122	0.120	0.122	0.119	0.109	0.332	0.330	0.332	0.325	0.310
		Hexaethylene glycol					Hexaethylene glycol				
Unmod	SPC/E	0.006	0.007	0.006	0.010	0.008	0.146	0.145	0.140	0.142	0.134
Unmod	TIP4P	0.006	0.006	0.008	0.009	0.008	0.146	0.143	0.140	0.137	0.131
Mod	SPC/E	0.003	0.003	0.003	0.002	0.002	0.075	0.075	0.075	0.070	0.072
Mod	TIP4P	0.003	0.002	0.003	0.003	0.003	0.075	0.073	0.074	0.074	0.074
		Intermolecular OH-OH					Intermolecular OH-OE				
		Diethylene glycol					Diethylene glycol				
Unmod	SPC/E	1.085	1.092	1.075	1.058	1.022	0.114	0.142	0.140	0.141	0.138
Unmod	TIP4P	1.085	1.093	1.077	1.058	1.022	0.114	0.142	0.140	0.140	0.139
Mod	SPC/E	0.829	0.825	0.817	0.805	0.788	0.089	0.089	0.089	0.088	0.088
Mod	TIP4P	0.829	0.824	0.818	0.808	0.791	0.089	0.089	0.088	0.086	0.088
		Tetraethylene glycol					Tetraethylene glycol				
Unmod	SPC/E	0.710	0.633	0.620	0.605	0.584	0.444	0.378	0.365	0.348	0.341
Unmod	TIP4P	0.710	0.628	0.618	0.612	0.584	0.444	0.379	0.362	0.361	0.348
Mod	SPC/E	0.164	0.164	0.163	0.168	0.160	0.064	0.080	0.063	0.070	0.063
Mod	TIP4P	0.164	0.165	0.160	0.159	0.162	0.064	0.066	0.059	0.061	0.064
		Hexaethylene glycol					Hexaethylene glycol				
Unmod	SPC/E	0.520	0.515	0.507	0.489	0.467	0.498	0.497	0.479	0.452	0.410
Unmod	TIP4P	0.520	0.518	0.505	0.492	0.469	0.498	0.499	0.485	0.461	0.422
Mod	SPC/E	0.138	0.138	0.136	0.134	0.131	0.166	0.166	0.159	0.157	0.143
Mod	TIP4P	0.138	0.138	0.136	0.134	0.130	0.166	0.168	0.160	0.153	0.139

[a] The specific TIP4P model used was TIP4P/2005

Table S4: Simulation results at 328K for end-to-end distances and radii of gyration

		w_{water}					w_{water}				
OPLS	Model[a]	0^2	0.001	0.005	0.010	0.020	0^2	0.001	0.005	0.010	0.020
		End-to-end distances/nm					Radii of gyration/nm				
		Diethylene glycol					Diethylene glycol				
Unmod	SPC/E	0.5672	0.5563	0.5563	0.5562	0.5555	0.2254	0.2237	0.2237	0.2237	0.2236
Unmod	TIP4P	0.5672	0.5563	0.5559	0.5553	0.5544	0.2254	0.2237	0.2236	0.2235	0.2234
Mod	SPC/E	0.4746	0.4742	0.4741	0.4740	0.4740	0.2112	0.2112	0.2112	0.2112	0.2112
Mod	TIP4P	0.4746	0.4742	0.4741	0.4739	0.4738	0.2112	0.2112	0.2112	0.2111	0.2111
		Tetraethylene glycol					Tetraethylene glycol				
Unmod	SPC/E	0.8163	0.8181	0.8152	0.8123	0.8052	0.3415	0.3418	0.3414	0.3411	0.3403
Unmod	TIP4P	0.8163	0.8171	0.8149	0.8110	0.8036	0.3415	0.3416	0.3414	0.3409	0.3401
Mod	SPC/E	0.6715	0.6676	0.6658	0.6634	0.6612	0.3209	0.3205	0.3204	0.3203	0.3203
Mod	TIP4P	0.6715	0.6670	0.6669	0.6648	0.6625	0.3209	0.3204	0.3205	0.3204	0.3204
		Hexaethylene glycol					Hexaethylene glycol				
Unmod	SPC/E	1.1765	1.1729	1.1639	1.1485	1.1216	0.4638	0.4633	0.4618	0.4594	0.4549
Unmod	TIP4P	1.1765	1.1715	1.1622	1.1497	1.1225	0.4638	0.4630	0.4616	0.4595	0.4551
Mod	SPC/E	1.1487	1.1428	1.1329	1.1231	1.1054	0.4619	0.4609	0.4591	0.4574	0.4542
Mod	TIP4P	1.1487	1.1431	1.1340	1.1232	1.1027	0.4619	0.4610	0.4593	0.4575	0.4538

[a] The specific TIP4P model used was TIP4P/2005

References cited in the Supportive Information

1. Edward, J. T., Molecular Volumes and the Stokes-Einstein Equation. *J. Chem. Educ.* **1970**, *47*, 261-270.
2. Hoffmann, M. M.; Too, M. D.; Paddock, N. A.; Horstmann, R.; Kloth, S.; Vogel, M.; Buntkowsky, G., On the Behavior of the Ethylene Glycol Components of Polydisperse Polyethylene Glycol PEG200. *J. Phys. Chem. B* **2023**, *127*, 1178−1196.
3. Hoffmann, M. M.; Horowitz, R. H.; Gutmann, T.; Buntkowsky, G., Densities, Viscosities, and Self-Diffusion Coefficients of Ethylene Glycol Oligomers. *J. Chem. Eng. Data* **2021**, *66*, 2480-2500.
4. Hoffmann, M. M.; Kealy, J. D.; Gutmann, T.; Buntkowsky, G., Densities, Viscosities, and Self-Diffusion Coefficients of Several Polyethylene Glycols. *J. Chem. Eng. Data* **2021**, *67*, 88-103.
5. Pokorný, V.; Serra, P. B. P.; Fulem, M.; Lima, C. F. R. A. C.; Santos, L. M. N. B. F.; Růžička, K., Heat Capacity and Phase Behavior of Selected Oligo(Ethylene Glycol)S. *J. Chem. Eng. Data* **2019**, *64*, 2742-2749.
